# A novel treatment for metastatic lymph nodes using lymphatic delivery and photothermal therapy

**DOI:** 10.1038/srep45459

**Published:** 2017-04-03

**Authors:** Adewale O. Oladipo, Oluwatobi S. Oluwafemi, Sandile P. Songca, Ariunbuyan Sukhbaatar, Shiro Mori, Junnosuke Okajima, Atsuki Komiya, Shigenao Maruyama, Tetsuya Kodama

**Affiliations:** 1 Department of Applied Chemistry, University of Johannesburg, P.O. Box 17011, Doornfontein 2028, Johannesburg, South Africa; 2 Centre for Nanomaterials Science Research, University of Johannesburg, P.O. Box 17011, Doornfontein 2028, Johannesburg, South Africa; 3 Department of Chemistry, University of Zululand, PB X1001, KwaDlangezwa, 3886, South Africa; 4 Laboratory of Biomedical Engineering for Cancer, Graduate School of Biomedical Engineering, Tohoku University, 4-1 Seiryo, Aoba, Sendai 980-8575, Japan; 5 Biomedical Engineering Cancer Research Center, Graduate School of Biomedical Engineering, Tohoku University, 4-1 Seiryo, Aoba, Sendai 980-8575, Japan; 6 Department of Oral and Maxillofacial Surgery, Graduate School of Dentistry, Tohoku University, 4-1 Seiryo, Aoba, Sendai 980-8575, Japan; 7 Department of Oral and Maxillofacial Surgery, Tohoku University Hospital, 1-1 Seiryo, Aoba, Sendai 980-8575, Japan; 8 Institute of Fluid Science, Tohoku University, 2-1-1 Katahira, Aoba Ward, Sendai, Miyagi 980-8577, Japan

## Abstract

Systemic delivery of an anti-cancer agent often leads to only a small fraction of the administered dose accumulating in target sites. Delivering anti-cancer agents through the lymphatic network can achieve more efficient drug delivery for the treatment of lymph node metastasis. We show for the first time that polymeric gold nanorods (PAuNRs) can be delivered efficiently from an accessory axillary lymph node to a tumor-containing proper axillary lymph node, enabling effective treatment of lymph node metastasis. In a mouse model of metastasis, lymphatic spread of tumor was inhibited by lymphatic-delivered PAuNRs and near-infrared laser irradiation, with the skin temperature controlled by cooling. Unlike intravenous injection, lymphatic injection delivered PAuNRs at a high concentration within a short period. The results show that lymphatic administration has the potential to deliver anti-cancer agents to metastatic lymph nodes for inhibition of tumor growth and could be developed into a new therapeutic method.

Cancer is the leading cause of death in the world with about 37% of the prognosis related to the presence of metastases in tumor-draining lymph nodes (LNs)^1^. LN resection for breast cancer is a recognized treatment but is not without its drawbacks and inherent limitations^2^,^3^. Complete resection of metastatic LNs was thought to eliminate any possible reoccurrence; however, in more aggressive cases, the procedure may leave behind a few cancer cells capable of proliferation. Furthermore, the structural disruption associated with resection poses additional risks such as seroma, numbness, lymphedema and infections^4^,^5^. To reduce these adverse effects, sentinel LN (SLN) dissection was developed as an alternative to traditional axillary LN dissection^6^. The surgical removal of a few regional LNs, thought most likely to contain cancer cells, does indeed provide some improvement. However, the prospect of residual microtumors and recurrence cannot be ruled out^7^. The use of chemotherapy and radiotherapy as standard clinical treatments for SLN metastasis has been explored^7^,^8^,^9^,^10^. These therapeutic techniques are associated with serious adverse effects on the patient’s postoperative quality of life as well as high treatment costs. In addition, the low absorption of anti-cancer agents after their systemic administration makes sufficient accumulation at the target site difficult, resulting in poor efficacy of the therapy. Hence, the development of a new, non-invasive, and effective treatment method would be highly desirable.

The triangular network formed by the lymphatic vessels connecting the subiliac LN (SiLN), proper axillary LN (PALN) and accessory axillary LN (AALN) has been studied and proposed to be an efficient route for drug delivery^11^,^12^. In addition, the emergence of photothermal therapy (PTT) as a minimally invasive therapeutic strategy has attracted interest in the field of nanoparticle-mediated cancer treatment. The local application of external near-infrared (NIR) laser radiation to nanoparticles causes strong light absorption and subsequent rapid and efficient conversion of photon energy into heat, leading to irreversible thermal damage to proteins and DNA^13^,^14^,^15^,^16^.

In our previous study in a mouse model, we demonstrated the fluid drainage routes between LNs and connecting lymphatic vessels and the potential of these routes as lymphatic drug delivery channels for use before surgery^11^,^12^. While the concept of using this delivery route has been highlighted, lymphatic delivery of an anti-cancer drug has never been reported. In the present study, we use an *in vivo* mouse model of LN metastasis to demonstrate the lymphatic delivery of polyethylene glycol (PEG)-modified gold nanorods (PAuNRs) and investigate the effects of NIR laser irradiation on tumor growth in the PALN (Fig. 1A). NIR laser irradiation to the metastatic PALN was commenced 160 s after the injection of PAuNRs into the AALN. To treat deeply embedded tumor cells and prevent skin burns, the laser intensity needed for hyperthermia was adjusted to heat the PALN to 45 °C while the surface temperature of the overlying skin was cooled with water (Fig. 1B). This procedure differs from established local/systemic delivery methods as it can deliver a high concentration of gold nanorods (AuNRs) to metastatic LNs within a short time.

## Results

### PAuNRs as an anti-cancer agent

**Figure 2:**
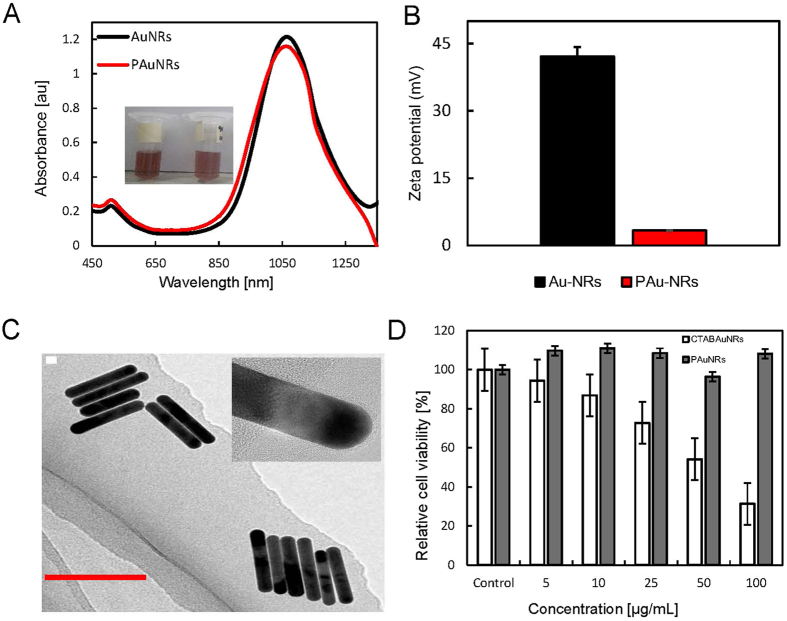
Characteristics of PAuNRs and *in vitro* toxicity. (**A**) Ultraviolet-visible-near infrared spectra of gold nanorods (AuNRs) before and after functionalization with thiol-terminated polyethylene glycol molecules (mPEG-SH). The inset shows photographic images of hexadecyltrimethylammonium bromide/sodium oleate (CTAB/NaOL) + AuNRs and polyethylene glycol (PEG)-modified AuNRs (PAuNRs). (**B**) Zeta-potentials of AuNRs with different surface modifications. CTAB/NaOL + AuNRs showed a positive charge; after PEGylation, the surface charge was close to neutral confirming the successful displacement of CTAB/NaOL by PEG molecules. (**C**) Transmission electron microscopy (TEM) image of monodispersed CTAB/NaOL + AuNRs. The inset shows a high resolution TEM image of AuNRs with a PEG coating. (**D**) Results of *in vitro* cytotoxicity assays in which FM3A-Luc cells were incubated with different concentrations of CTAB/NaOL + AuNRs or PAuNRs for 24 h. Data are presented as mean ± SD (*n* = 3).

PEGylated AuNRs with a longitudinal surface plasmon resonance of 1066 nm were successfully prepared via the modified procedure we proposed (see Methods). The thiol-terminated PEG molecules (mPEG-SH) displaced the hexadecyltrimethylammonium bromide (CTAB) surfactant on the AuNRs surface, thereby eliminating the inherent toxicity usually conferred by CTAB. In addition, the use of mPEG-SH enhances the circulation of AuNRs and improves the passive targeting of tumor cells by AuNRs due to the enhanced permeability and retention (EPR) effect^15^. The ultraviolet (UV)-visible (VIS)-NIR spectra of both CTAB-coated AuNRs and PAuNRs are shown in Fig. 2A. The spectra confirmed efficient surface functionalization of the AuNRs surface by mPEG-SH without any change in the localized surface plasmon resonance wavelength. However, there was a change in the surface charge. The CTAB/sodium oleate (NaOL)-coated AuNRs showed a positive zeta-potential (+42.13 mV), while attachment of PEG to the AuNRs was demonstrated by a reduction in zeta-potential to a near neutral charge (+3.15 mV) (Fig. 2B). Transmission electron microscopy (TEM) images (Fig. 2C) showed that the AuNRs were uniform and monodispersed with an average diameter of 10.1 ± 0.35 nm and an aspect ratio of 6.71. Prior to its use for *in vivo* applications, the cellular cytotoxicity of PAuNRs on FM3A-Luc mammary carcinoma cells was examined. FM3A-Luc cells treated with PAuNRs for 24 h maintained a high cell viability (above 90%), in contrast to those treated with CTAB/NaOL + AuNRs, suggesting negligible toxicity (Fig. 2D).

### *In vivo* NIR fluorescence imaging and evaluation of biodistribution

**Figure 3:**
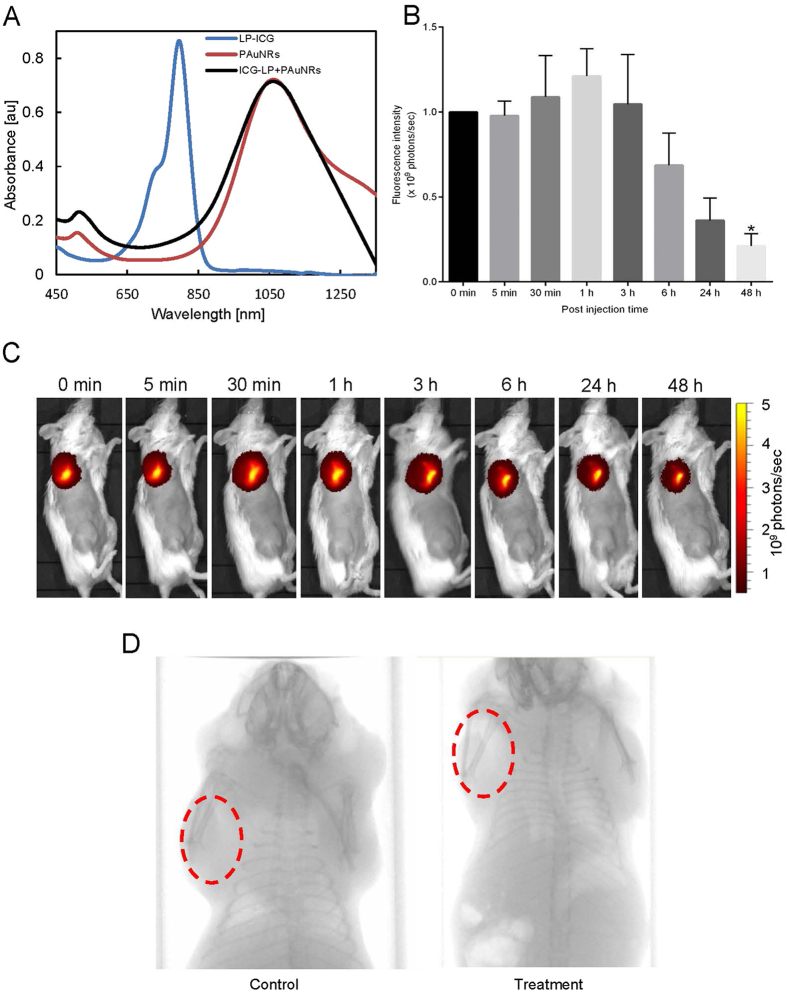
Time course of the *in vivo* biodistribution of gold nanorods conjugated with indocyanine green-liposomes (ICG-LP + PAuNRs) (**A**) Ultraviolet-visible-near infrared (NIR) spectra of indocyanine green-liposomes (ICG-LP), hexadecyltrimethylammonium bromide/sodium oleate + gold nanorods (CTAB/NaOL + AuNRs), and polyethylene glycol-modified gold nanorods conjugated with ICG-LP (ICG-LP + PAuNRs). (**B**) Normalized fluorescence signal counts in the axillary area of mice at various time points after the injection of 120 μL of ICG-LP + PAuNRs into the accessory axillary lymph node (AALN). Fluorescence counts are shown as the mean ± SEM (*n *=* *3). Statistical significance is indicated by *(*P* < 0.05). (**C**) NIR fluorescence images of the axillary region of a mouse taken at various times after the injection of 120 μL of ICG-LP + PAuNRs into the AALN. (**D**) X-ray micro-computed tomography imaging of the proper axillary lymph node (PALN) before and 3 min after the injection of PAuNRs into the AALN.

A fluorescent gold nanorods conjugated with indocyanine green-liposomes (ICG-LP + PAuNRs) was synthesized (Fig. 3A) and its *in vivo* biodistribution was assessed. The axillary area of each mouse was imaged before treatment and at various time intervals after the injection of ICG-LP + PAuNRs into the AALN (*n *=* *3). We observed rapid flow of ICG-LP + PAuNRs into the PALN within 2 min after injection, which is consistent with our earlier study^11^. The metastatic PALN had completely filled with ICG-LP + PAuNRs at 30 min after injection, and the fluorescence intensity continued to increase up to 1 h after injection as more of the nanoprobe arrived from the AALN (Fig. 3B). From 1 h after injection onwards there was a gradual clearance of ICG-LP + PAuNRs from the PALN, as indicated by a decrease in the fluorescence intensity. Minimal residual fluorescence was observed after 24 h with a statistical difference (*P < 0.05). This suggests a long circulation time for ICG-LP + PAuNRs within the tumor vasculature, indicating that a long time window is available for an efficient thermal procedure (Fig. 3C). X-ray micro-computed tomography (CT) imaging of the axillary regions of mice before and 2 min after the injection of PAuNRs further confirmed the accumulation of PAuNRs in the PALN (Fig. 3D). The post-injection image showed enhanced contrast compared to the pre-injection image. We attribute this change to the enhanced plasmon absorption and scattering properties of PAuNRs that had accumulated in the PALN. Next, we investigated the *ex vivo* biodistribution profiles in organs harvested 24 h and 48 h after injection of the nanoprobe. High retention of ICG-LP + PAuNRs in the AALN was observed 24 h after their lymphatic administration, but the levels in the AALN after 48 h were lower due to the probe draining out into the PALN. These results were found to be consistent with the hypothesized EPR effect of the drug/nanocarrier within the tumor space due to perfusion. We also noticed significant accumulation of the probe in highly vascularized tissues (liver and spleen) both early after the administration and at 48 h post-injection (see Supplementary Fig. S1).

### Anti-tumor effect of PTT using PAuNRs

**Figure 4:**
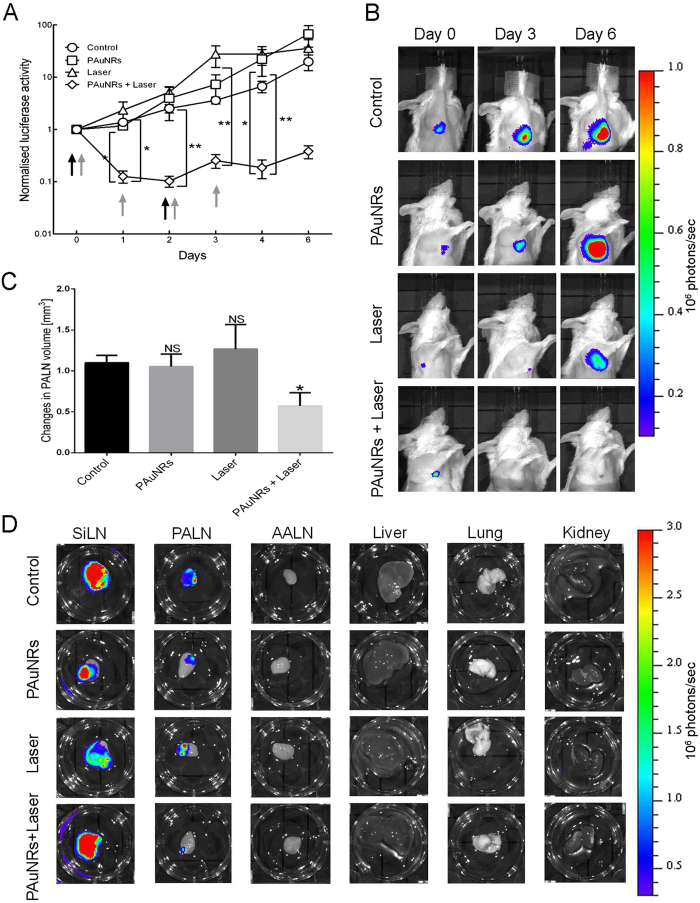
Anti-tumor effects of various treatment methods. (**A**) Normalized luciferase activity in the metastatic proper axillary lymph nodes (PALNs) of mice in the Control, PAuNRs, Laser and PAuNRs + Laser groups. Quantified data are presented as the mean ± SEM (*n *=* *5). **P < 0.01, *P < 0.05; one-way ANOVA. The black arrow indicates the day of injection of polyethylene glycol-modified gold nanorods (PAuNRs) or phosphate-buffered saline (PBS), while the grey arrow indicates the day of laser irradiation. (**B**) Representative bioluminescence images acquired using an *in vivo* bioluminescence imaging system showing the tumor activity in the metastatic PALN on treatment days 0, 3 and 6. The first dose of the treatment agent was administered on day 0. (**C**) Volume changes of the tumor-bearing PALN between day 0 and day 6 of treatment, assessed using three-dimensional high-frequency ultrasound. The values were normalized to those on day 0 and are expressed as mean ± SEM (*n *=* *5). *P < 0.05 versus the Control group. (**D**) Representative *ex vivo* bioluminescence images of tumor in organs harvested on day 6 after treatment.

Clinical studies have demonstrated that PTT, using both local and systemic delivery, is a non-invasive, selective, and efficient therapy for various types of cancer. In the present study, we evaluated the *in vivo* anti-cancer efficacy of PTT over a 6-day period, with PAuNRs (120 μL of 40 μg/mL) delivered via the lymphatic system. After confirming the presence of metastasis in the PALN, the AALN of the mouse was injected on day 0 and day 2 with either PAuNRs or phosphate-buffered saline (PBS, as a control). Four experimental groups were used. Mice in the Control group (AALN injected with PBS) and PAuNRs group (AALN injected with PAuNRs) received no irradiation with laser. For mice in the Laser group, the AALN was injected with PBS and the PALN was irradiated on days 0, 1, 2 and 3. For mice in the PAuNRs + Laser group, the AALN was injected with PAuNRs and the PALN was irradiated on days 0, 1, 2 and 3. Laser irradiation of the PALN was commenced 160 s after the injection of PAuNRs into the AALN and after the surface of the skin overlying the PALN had been water-cooled to <15 °C using a temperature control system. The local rise in temperature in the PALN induced by laser irradiation was increased by the presence of PAuNRs that had been administered via lymphatic delivery. Maintenance of the temperature of the PALN at 45 °C for 5 min (under controlled skin surface cooling) required a laser power of 2.55 ± 0.45 W with PAuNRs and 3.16 ± 0.33 W without PAuNRs (see Supplementary Fig. S2). The tumor activities in the PALNs of mice in each group were determined daily for 6 days. The progression of tumor growth was assessed using *in vivo* bioluminescence imaging (IVIS) and a high-frequency ultrasound system (VEVO). Figure 4 shows the change in the luciferase activity of the PALN with time. We observed no significant anti-tumor effect in the Control and PAuNRs groups (which were not irradiated), as tumor growth (measured as luciferase activity in the PALN) increased steadily with time. Similarly, PALN luciferase activity continued increasing with time in the Laser group despite irradiation, indicating continuous tumor growth within this LN. However, we noticed a significant decrease in PALN luciferase activity in the PAuNRs + Laser group after treatment on day 1, which was maintained until day 6 (Fig. 4A). Tumor growth was terminated only in the PAuNRs + Laser group, indicating a disruption of tumor perfusion (Fig. 4A and B). These results suggest that the photothermal effects of irradiation alone or PAuNRs alone were not sufficient to inhibit tumor progression. We anticipated that after the first injection of PAuNRs, the residual content of nanorods after 24 h would be sufficient for another laser irradiation without requiring the injection of additional hyperthermia agent. This was made possible by the good accumulation and retention of PAuNRs within the tumor mass afforded by this delivery route. The good retention properties made manipulation of the treatment conditions and timing possible. As shown in Fig. 4C, the PALN volume almost doubled during the treatment period in the Control, PAuNRs and Laser groups, indicating a continuous proliferation of tumor cells in the PALN. In contrast, there was a significantly smaller increase in PALN volume in the PAuNRs + Laser group than in the other 3 groups (*P* < 0.05). The smaller increase in PALN volume in the PAuNRs + Laser group was consistent with the observed decrease in luciferase activity. *Ex vivo* bioluminescence imaging of harvested tissues further revealed a notable tumor burden in the PALN for the Control, PAuNRs and Laser groups, whereas we did not notice any light intensity arising from tumor activity in the PALN for the PAuNRs + Laser group (Fig. 4D).

### Morphological changes in the metastatic PALN

**Figure 5:**
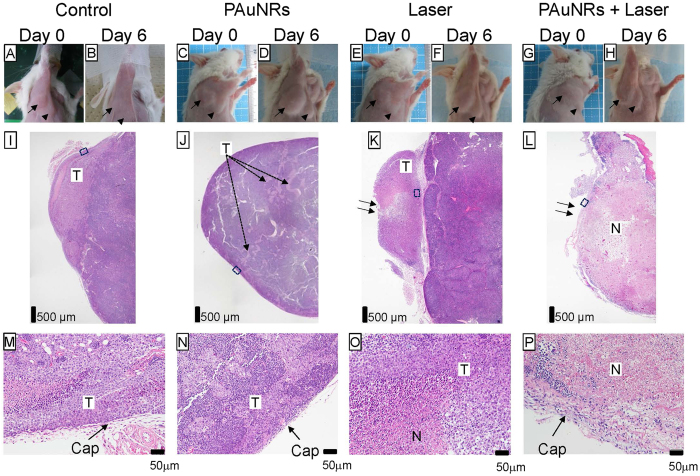
Histological analysis of tumor-bearing lymph nodes. **(A,B,I,M)**: control. **(C,D,J,N)**: PAuNRs. **(E,F,K,O)**: Laser. **(G,H,L,P)**: PAuNRs + Laser. **(A,C,E,G)**: Day 0, before treatment. **(B,D,F,H)**: Day 6, after treatment. Arrows in (**A**–**H**) highlight the accessory axillary lymph node (AALN). Arrowheads in A-H indicate the proper axillary lymph node (PALN). Representative of *n *=* *20. (**I**–**P**) Hemotoxylin and eosin (HE) staining of the PALN at Day 6. (**I**) Tumor cells had metastasized and invaded into the cortex region. (**I**–**L**) Representative sections of the PALN in mice from the Control (*n *=* *5), PAuNRs (*n *=* *5), Laser (*n *=* *5) and PAuNRs + Laser (*n *=* *5) groups. Scale bars: 500 μm. Arrows highlight the direction of the radiation beam. Boxes in (**I**–**L**) outline the magnified views shown in (**M**–**P**). Scale bars: 50 μm (magnified view). Arrows highlight the capsule of each PALN. N: necrotic area; T: tumor area; Cap: capsule.

In order to detect skin burn caused by radiation in the region of the PALN, we captured images before (Day 0) and after (Day 6) each treatment (Fig. 5). Macroscopic images obtained on Day 6 after laser treatment (Fig. 5F and H) indicated successful cooling of the skin surface by the temperature cooling system, as was also demonstrated in our previous study^16^. Hematoxylin and eosin (HE) staining was carried out to evaluate tissue damage in the PALN on Day 6 (Fig. 5I–P). Representative images of a section of a whole LN are shown in Fig. 5I–L. Areas of tumor were detected in the Control and PAuNRs groups, while areas of necrosis were detected in the Laser group (Fig. 5K) and PAuNRs + Laser group (Fig. 5L). In magnified views of the PALN (Fig. 5M–P), extracapsular spread of metastasis and some necrotic areas were observed in the Laser group (Fig. 5K and O). Capsule damage was not detected in the Laser group (Fig. 5K and O) or PAuNRs + Laser group (Fig. 5L and P). These results indicate that the combination of PAuNRs (delivered via the lymphatic system) and laser irradiation (with skin surface cooling) has the highest anti-tumor potential and the lowest risk of morphological changes, as compared with irradiation alone or PAuNRs alone.

## Discussion

This study is the first report that demonstrates the use of photothermal therapy for the treatment of metastatic lymph nodes (PALN) using an anti-cancer agent (PAuNRs) delivered via a lymphatic route. In our model of LN metastasis, tumor cells were inoculated into the SiLN to induce metastasis in the PALN via lymphatic vessels, and PAuNRs were injected into the AALN to deliver them to the PALN. Although the mechanism underlying metastasis in this model differs from that underlying metastasis from a solid tumor in a physiological system, the reproducibility of the model allowed us to focus on the main aim of our study, namely to investigate the therapeutic effects of combining PAuNRs delivered by a lymphatic drug delivery system with NIR laser light irradiation. Since this was a proof-of-concept study, changes in the microenvironment such as the development of lymphatic niches were not considered. It will be important to validate our findings in future studies using spontaneous cancer models.

Accumulation of PAuNRs within the metastatic PALN is crucial for successful tumor ablation by PTT in future biomedical application. The ability of PAuNRs to passively target, accumulate and remain in the PALN at a high concentration for a long period, and evade immune clearance, makes them desirable. PEG-modified AuNRs, which showed minimal cytotoxicity and passive targeting ability, were employed as a biocompatible hyperthermia agent. The biocompatibility of the PAuNRs was confirmed by a near-neutral surface charge indicated by the zeta-potential. When a solution of the PAuNRs was injected into the AALN, it reached the PALN successfully and penetrated within the tumor vasculature of the metastatic PALN^12^. The encapsulation of indocyanine green (ICG) in liposomes enhances the fluorophore of the ICG-LP + PAuNRs nanoprobe synthesized. This increases its stability after injection, thus allowing real-time imaging of the nanoprobe for effective monitoring of the biodistribution of the PAuNRs. The results from fluorescence imaging experiments suggest that a substantial proportion of the total PAuNRs dose administered accumulated in the target site, rather than at non-target organs such as the spleen and liver, as early as 3 min post-injection (Fig. 3B and C). The anti-tumor effect of combining lymphatic PAuNRs administration with NIR laser irradiation was higher than that achieved with NIR laser irradiation or PAuNRs alone, as demonstrated by the luciferase activity changes in the metastatic PALN (Fig. 4A and B). In addition, the use of a lower laser power (2.55 ± 0.45 W) to maintain temperature at 45 °C further confirmed successful accumulation of PAuNRs in the tumor-bearing PALN. A higher laser power (3.16 ± 0.33 W) was required to maintain a similar temperature when no PAuNRs were injected (see Supplementary Fig. S2). A sensitive method for assessing the anti-tumor effect is the *ex vivo* evaluation of tumor load, which showed no luciferase activity in the harvested PALN of the PAuNRs + Laser group. An interesting feature in this study is the absence of distant metastasis in the organ (lungs) closest to the metastatic area as shown in Fig. 4D. These results suggest that laser irradiation after lymphatic delivery of PAuNRs led to necrosis of the tumor mass within the PALN. A significant treatment effect was achieved largely due to sufficient accumulation of PAuNRs in the PALN via lymphatic delivery. The evaluation of tissue damage induced by this treatment method was subsequently performed by histological analysis. Irradiation in the Laser group showed no obvious laser-induced skin burn injuries in all mice. Tumor cells were destroyed with no tumor regrowth observed after treatment, as no viable tumor cells were found. In a previous study^14^, we investigated photothermal therapy of tumors in LNs using near-infrared laser light and various types of AuNRs, including bare AuNRs, neutravidin polymer-conjugated AuNRs and fluorescent AuNRs conjugated with Atto 590-biotin. There were no significant differences in body weight and serum total bilirubin, alanine aminotransferase, aspartate aminotransferase and blood urea nitrogen between control mice and those administered AuNRs, suggesting no major systemic toxicity. Furthermore, the administration of AuNRs using a lymphatic drug delivery system has been shown to reduce acute toxicity compared with intravenous injection^17^,^18^. In the present study, macroscopic observations and histological analysis showed no evidence of severe damage caused by PAuNRs. Taken together, these findings suggest that PAuNRs do not exert notable toxicity, although additional studies are needed to determine their safety profile before this research can be translated to the clinical setting.

In summary, we have reported for the first time the successful use of a novel PTT technique, involving delivery of PAuNRs through a lymphatic route and NIR irradiation, which showed therapeutic efficacy against LN metastasis. Unlike previous methods utilizing conventional systemic/local delivery of hyperthermia agents to target sites, lymphatic-delivered PAuNRs completely filled the metastatic region at a high concentration within a short period. The use of this delivery route leads to prolonged accumulation and retention of PAuNRs within the target site, allowing for precise and flexible control of the treatment conditions and time course. The improved accumulation of PAuNRs in the tumor facilitated the hyperthermic effects of NIR laser irradiation, thereby inhibiting tumor growth. This finding shows the tremendous potential of this delivery route in the treatment of metastatic LNs. Furthermore, the results show that lymphatic delivery of PAuNRs combined with NIR laser-induced thermolysis could achieve targeted treatment of metastasis. Therefore, we believe that this novel antitumor technique will provide an alternative approach for the efficient treatment of metastasis.

## Methods

Animal experiments were carried out in accordance with the approved guidelines set out by the Institutional Animal Care and Use Committee of Tohoku University.

### PEGylated gold nanorod synthesis

High aspect ratio AuNRs were synthesized using a seed-mediated technique using the method of Ye *et al*.^19^, with slight modifications. In a typical reaction, 0.3645 g CTAB was added to 10 mL water and heated to 40 °C to completely dissolve the powder. 0.250 mL of HAuCl_4_ solution (0.01 M) was added to the CTAB solution and stirred gently for 30 min. To this solution, a freshly prepared 0.6 mL aliquot of 0.01 M NaBH_4_ was added under vigorous stirring for 2 min to produce a light brown solution that served as the seed solution. The solution was kept at 30 °C for 30 min before use. To prepare the growth solution, 0.037 M of CTAB and 1.234 g NaOL were dissolved in 250 mL of warm water (~50 °C) in a 1 L Erlenmeyer flask. The solution was allowed to cool down to 30 °C and 4 mM AgNO_3_ solution, in various volumes, was added. The mixture was left undisturbed at 30 °C for 15 min after which 250 mL of 1 mM HAuCl_4_ solution was added. The solution became colorless after 60 min of stirring (700 rpm) and a minimum volume of HCl was then introduced to adjust the pH. After another 15 min of slow stirring at 400 rpm, 1.25 mL of 0.064 M ascorbic acid (AA) was added and the solution was vigorously stirred for 30 s. Finally, a small amount of seed solution was injected into the growth solution. The resultant mixture was stirred for 30 s and left undisturbed at 30 °C for 12 h to allow nanorod growth to occur. The final products were isolated by centrifugation at 7,000 rpm for 30 min followed by removal of the supernatant. No size and/or shape-selective fractionations were performed. Before functionalization with mPEG-SH (MW 5,000), 500 μL of the nanorods were centrifuged at 8,000 rpm for 10 min to remove any excess CTAB. This was followed by the addition of 500 μL of 1 mM mPEG-SH and 100 μL 1X–PBS under magnetic stirring for 24 h. Finally, the mPEG-SH functionalized AuNRs were obtained after centrifugation to remove any unbounded mPEG-SH and sonicated for 10 min. The nanorods were re-dispersed in 1X–PBS solution for further analysis and characterization.

### Characterization of AuNRs

Absorption spectra were primarily obtained using a JASCO V-770 NIR spectrophotometer. TEM was performed using a JEOL JEM-2100F HRTEM at an accelerating voltage of 200 kV. Zeta potential measurements were made using a Photal ELS-Z2MH instrument. Centrifugation was carried out using an Eppendorf 5415 R and KUBOTA 7780II centrifuge.

### Cell culture

C3H/He mouse mammary carcinoma cells (FM3A-Luc)^20^, which stably express a firefly luciferase gene, were used after being passaged three times. Cells were cultivated in RPMI-1640 medium (Sigma-Aldrich) supplemented with 10% heat-inactivated fetal bovine serum, 0.5% Geneticin G418 and 1% L-glutamine–penicillin–streptomycin (Sigma-Aldrich). Cells were incubated at 37 °C in a mixture of 5% carbon dioxide and 95% air until 80% confluence was achieved. On the day of inoculation, the absence of *Mycoplasma* contamination was confirmed using a MycoAlert *Mycoplasma* Detection Kit (Lonza Rockland), according to the manufacturer’s protocol.

### Cell viability assay

Cells were maintained in RPMI-1640 medium supplemented with 10% heat-inactivated fetal bovine serum, 0.5% Geneticin G418 and 1% L-glutamine–penicillin–streptomycin (Sigma-Aldrich). For incubation with PAuNRs, the cells were plated in triplicate at a density of 5,000 cells/well in 48-well microplates. After 24 h incubation (37 °C, 5% carbon dioxide), cells were exposed to PAuNRs at different concentrations (diluted in 10 μL of medium) in triplicate for 1 h. The medium was removed and the cells washed twice with PBS. Fresh medium was added (500 μL/well) and the plate incubated for 24 h. Cytotoxicity was assessed using a standard MTT method.

### Mice

MXH10/Mo-*lpr*/*lpr* (MXH10/Mo/lpr) mice (12–14 weeks of age)^20^ were bred under specific pathogen-free conditions in the Animal Research Institute, Graduate School of Medicine, Tohoku University, Sendai, Miyagi, Japan. MXH10/Mo/lpr mice develop systemic swelling of lymph nodes up to 10 mm in diameter at 2.5–3 months of age through the accumulation of a large number of lpr-T cells in the lymph nodes^20^. These mice do not express the Fas gene involved in apoptosis^21^. The basic internal structure of the lymph nodes is preserved in these mice, including the structures of the cortex, paracortex and medullary regions^20^.

### Induction of metastasis in the PALN by injection of tumor cells into the SiLN

Metastasis to the PALN was induced by injecting 3.3 × 10^5^ cells suspended in a mixture of 20 μL PBS and 40 μL of 400 mg/mL Matrigel (Collaborative Biomedical Products) into the unilateral SiLN (*n* = 23). Intranodal inoculation into the SiLN was carried out using a 27 G needle under the direction of a high-frequency ultrasound imaging system (VEVO770; VisualSonics) with a 25-MHz transducer (RMV-710B; axial resolution, 70 μm; focal length, 15 mm; VisualSonics). The needle was maintained in the same position for 1 min to solidify the Matrigel after removal of the needle. The inoculation day was defined as day 0.

### Tumor growth and metastasis detection by *in vivo* bioluminescence imaging

Metastasis to the PALN was assessed using an *in vivo* bioluminescence imaging system (IVIS Lumina; PerkinElmer) every 3 days post-inoculation. The background luciferase activity was ~4 × 10^4^ photons/sec. Mice whose luciferase activity in the PALN was larger than the background level (~5 × 10^5^ photons/sec) were considered as being metastatic mice. The probability of metastasis occurring in the PALN was about 87% (20 events/23 mice). 150 mg/kg luciferin (Promega) was injected intraperitoneally. After 10 min, luciferase bioluminescence was measured for 1 min, using IVIS. The metastatic mice were divided into four groups: Control group (*n *=* *5), PAuNRs group (*n *=* *5), Laser group (*n *=* *6) and PAuNRs + Laser group (*n *=* *7).

### Laser irradiation of metastatic PALNs

PTT with PAuNRs was performed using *in vivo* laser irradiation. The anti-tumor effects of PTT on malignant cells was evaluated using a continuous wave Nd:YVO_4_ air-cooled NIR laser (2.5 ± 0.5 W/cm^2^; wavelength, 1064 nm; TEM_00_ beam diameter, 0.6 mm; CYD-010-TUBC, Neoarc). PAuNRs (40 μg/mL, 120 μL) were intranodally injected into the AALNs of the tumor-bearing MXH10/Mo/lpr mice. The skin temperature was cooled to <15 °C before irradiation by the temperature control system (cooling system water temperature, 10 °C; water flow rate, 760 mL/sec)^15^ and the irradiated site temperature was measured using a K-type thermocouple (Ishikawa Trading)^16^. The PALNs of the mice were exposed to the laser (1064 nm) 160 s post-injection of PAuNRs, with the laser focused on a spot (up to 6 mm in diameter) at the target site. The temperature of the PALN was maintained at 45 °C for 5 min by tuning the intensity of the laser beam.

### *In vivo* anti-tumor effects

The anti-tumor efficacies of the various treatment procedures were evaluated by measuring the luciferase activities of metastases in the PALNs on days 0, 1, 2, 3, 4 and 6 after treatment, using an *in vivo* bioluminescence system^22^. The first day of treatment was designated as day 0. To monitor effectively luciferase activity in the PALNs, the SiLNs were obscured with an aluminum foil and black cardboard. The PALN volume changes were measured on day 0 and day 6 with a high-frequency ultrasound system (VEVO770; VisualSonics)^23^,^24^.

### *In vivo* biodistribution of ICG-LP-conjugated PAuNRs

For the *in vivo* biodistribution of nanorods, mice were placed in a gas chamber and anesthetized using 2% isoflurane in oxygen. 120 μL of the ICG-LP + PAuNRs probe was injected into the AALNs. Then, the ICG-based fluorescence signal was recorded at different time intervals after injection with the IVIS (excitation filter set at ICG; emission wavelength of 745 nm; 1 sec exposure time).

### *Ex vivo* biodistribution of ICG-LP-conjugated PAuNRs

Mice were sacrificed and organs harvested 24 h or 48 h post-injection of ICG-LP + PAuNRs. Blood was drawn from the posterior vena cava. The organs and tissues were quickly imaged *ex vivo* based on the fluorescence signal of ICG.

### *Ex vivo* bioluminescence imaging

To assess the tumor burden in the PALN after treatment, harvested LNs, kidneys, lungs and liver were placed in 0.3 mg/mL of D-luciferin in PBS. The organs were immersed for 5 min before imaging at a 1 sec exposure time using IVIS. The region of interest (ROI) tool was used to quantify the luciferase activity from the individual organs to compare the extents of the tumor burden.

### Histological analysis

All the harvested samples were fixed in 10% formaldehyde in PBS (Rapid Fixative, Kojima Chemical Industry) for 4 days at 4 °C, dehydrated and embedded in paraffin. The embedded specimens were cut serially into 2–4 μm sections and stained with HE using an automated HE staining processor (Symphony; Ventana Medical Systems, Inc.). The specimen boundary was determined under low magnification using a BX-51 Olympus microscope connected to a digital camera (DP72; Olympus).

### Numerical analysis

Numerical analysis was carried out based on our previous report^16^.

### Statistical analysis

All measurements are presented as the mean ± SEM. Differences between groups were determined by one-way ANOVA followed by the Kruskal-Wallis test. A *P* value < 0.05 was considered to represent a statistically significant result. Statistical analyses were conducted using Excel 2010 (Microsoft) and GraphPad Prism software.

## Additional Information

**How to cite this article:** Oladipo, A. O. *et al*. A novel treatment for metastatic lymph nodes using lymphatic delivery and photothermal therapy. *Sci. Rep.* **7**, 45459; doi: 10.1038/srep45459 (2017).

**Publisher's note:** Springer Nature remains neutral with regard to jurisdictional claims in published maps and institutional affiliations.

## Supplementary Material

Supplementary Information

## Figures and Tables

**Figure 1:**
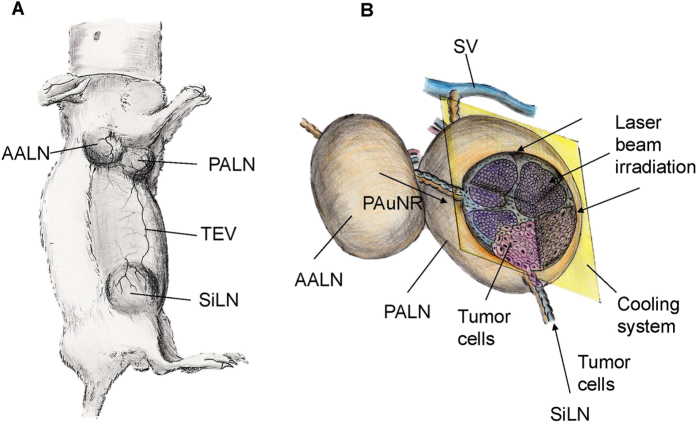
Illustration of the MXH10/Mo/lpr mouse and treatment approach. (**A**) MXH10/Mo/lpr mouse. AALN: accessory axillary lymph node; PALN: proper axillary lymph node; SiLN: subiliac lymph node; TEV: thoracoepigastric vein. (**B**) Treatment method. Tumor cells were inoculated into the subiliac lymph node (SiLN) to induce metastasis to the proper axillary lymph node (PALN). Polymeric gold nanorods (PAuNRs) were injected into the accessory axillary lymph node (AALN) after metastasis had been induced in the PALN. Laser irradiation of the PALN was carried out while controlling the overlying skin temperature with a water-cooling system. The near-infrared (NIR) laser beam (intensity: 2.55 ± 1.0 W/cm^2^; wavelength: 1064 nm) was applied for 300 sec.
