# Supplementary material for: A novel treatment for metastatic lymph nodes using lymphatic delivery and photothermal therapy
Source: Sci Rep. 2017 Apr 3;7:45459. doi: 10.1038/srep45459 (PMC5377366; doi:10.1038/srep45459)
Supplement: Supplementary Information [file srep45459-s1.pdf]

Supplementary Info File

A novel treatment for metastatic lymph nodes using lymphatic delivery and photothermal therapy

Adewale O. Oladipo[1,2], Oluwatobi S. Oluwafemi[1,2], Sandile P. Songca[3], Ariunbuyan Sukhbaatar[4], Shiro Mori[5,6], Junnosuke Okajima[7], Atsuki Komiya[7], Shigenao Maruyama[7], and Tetsuya Kodama[4,5]

Corresponding author:

Tetsuya Kodama, PhD (Eng.), PhD (Med.)

Laboratory of Biomedical Engineering for Cancer

Graduate School of Biomedical Engineering

Tohoku University, 4-1 Seiryo, Aoba, Sendai, Miyagi 980-8575, Japan.

Tel & Fax: +81-22-717-7583

E-mail: kodama@tohoku.ac.jp

Supplementary Figures

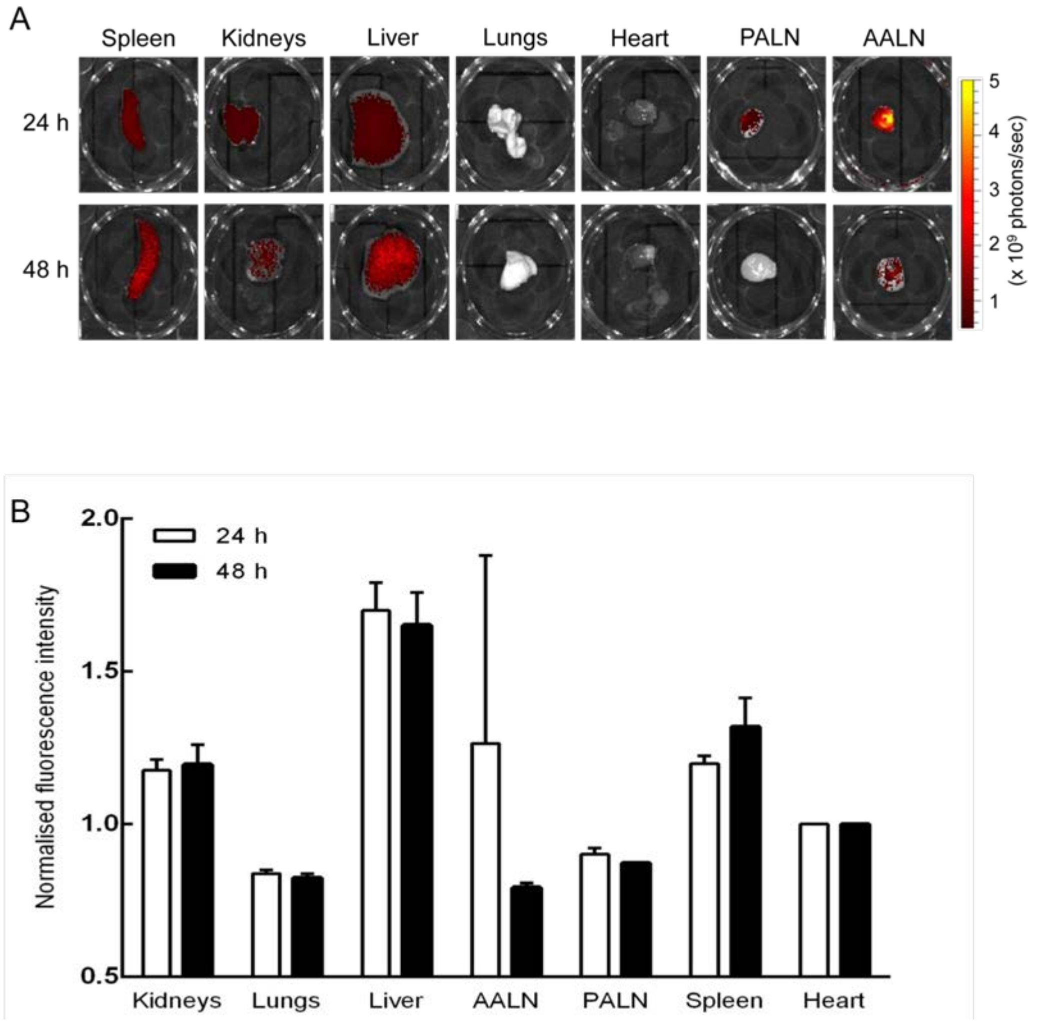

Supplementary Figure 1.

Ex vivo fluorescence imaging of the harvested organs.

A. *Ex vivo* assessment of the biodistribution of polyethylene glycol-modified gold nanorods conjugated with indocyanine green-liposomes (ICG-LP + PAuNRs) in various organs harvested 24 h and 48 h after injection.

B. Normalized fluorescence intensity values of various organs (normalized with respect to the heart) imaged 24 h and 48 h after the injection of ICG-LP + PAuNRs. Data are presented as the mean ± SD.

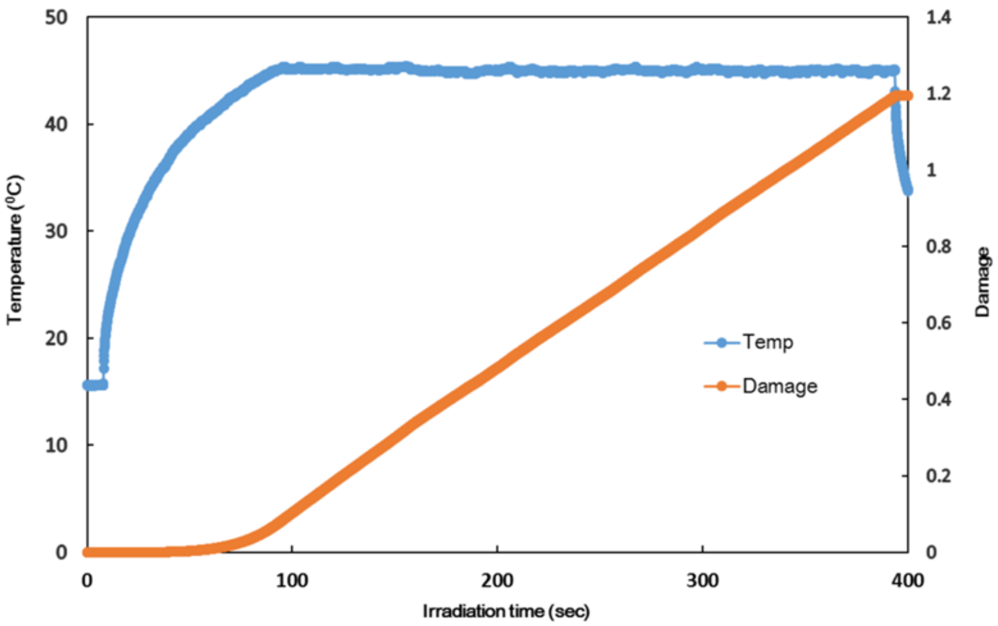

Supplementary Figure 2

Temperature changes in the PALN during its irradiation with near-infrared laser light, with use of the water-cooling system to control skin surface temperature. The blue line represents the temperature change in the PALN while the orange line represents the damage function for the PALN. The temperature in the PALN reached 45°C after approximately 100 sec of irradiation and was maintained throughout the remainder of the treatment period. A damage function of 1.2 at the end of the treatment period indicates no severe burn injury to the PALN surface after treatment.
